# Prize Essay on Vital Statistics

**Published:** 1867-09

**Authors:** 


Prize Essay on Vital Statistics. By FRANKLIN B. HOUGH, M.D., of Lowville, N. Y.

This is a pamphlet of 37 pages, being the Essay to which was awarded the Brinsmade prize of $100, by the New York State Medical Society, in February, 1867. The subject of which it treats is of great importance. One of the leading objects of the writer was to present a feasable plan for making and tabulating hospital reports, records of private practice in medicine, surgery, and obstetrics; together with the draft of a law for the registration of births, marriages, and deaths.

We have not had time to examine the plan presented, but will endeavor to refer to it again at a future time.

